# Genomic analysis of the progenitor strains of *Staphylococcus aureus* RN6390

**DOI:** 10.1099/acmi.0.000464.v3

**Published:** 2022-11-29

**Authors:** Stephen R. Garrett, Giuseppina Mariano, Tracy Palmer

**Affiliations:** ^1^​ Microbes in Health and Disease Theme, Newcastle University Biosciences Institute, Newcastle University, Newcastle upon Tyne, NE2 4HH, UK

**Keywords:** genome analysis, NCTC8325, RN6390, *Staphylococcus aureus*, type strain

## Abstract

RN6390 is a commonly used laboratory strain of *Staphylococcus aureus* derived from NCTC8325. In this study, we sequenced the RN6390 genome and compared it to available genome sequences for NCTC8325. We confirmed that three prophages, Φ11, Φ12 and Φ13, which are present in NCTC8325 are absent from the genome of RN6390, consistent with the successive curing events leading to the generation of this strain. However, we noted that a separate prophage is present in RN6390 that is not found in NCTC8325. Two separate genome sequences have been deposited for the parental strain, NCTC8325. Analysis revealed several differences between these sequences, in particular, between the copy number of *esaG* genes, which encode immunity proteins to the type VII secreted anti-bacterial toxin, EsaD. Single nucleotide polymorphisms were also detected in ribosomal RNA genes and genes encoding microbial surface components recognizing adhesive matrix molecules (MSCRAMM) between the two NCTC8325 sequences. Comparing each NCTC8325 sequence to other strains in the RN6390 lineage confirmed that sequence assembly errors in the earlier NCTC8325 sequence are the most likely explanation for most of the differences observed.

## Impact statement

RN6390 is a commonly used laboratory strain of *Staphylococcus aureus *, for which a genome sequence was not previously available. Several prior studies of RN6390 have utilized the genome of progenitor strain NCTC8325, sequenced by the University of Oklahoma, as a reference. In this study we highlight genetic differences between RN6390 and this progenitor sequence, noting several disparities in highly repetitive regions. Comparison of the RN6390 genome sequence with other strains in the same lineage and with a genome sequence of NCTC8325 sequenced independently by the Wellcome Sanger Institute indicates that assembly error rather than genetic changes accounts for the differences observed between RN6390 and the NCTC8325-Oklahoma sequence.

## Data summary

Whole-genome sequences of the bacterial strains used in this study are available on NCBI (RN6390: CP090001.1, RN25: JAJSOX000000000.1, RN450: JAJSOY000000000.1, ISP479c: JAMWMH000000000.1). SNP tables are available in Tables S1–3. All custom scripts are available on Github: https://github.com/GM110Z/Garret-et-al.-recombination-paper.

## Introduction

**Fig. 1.**
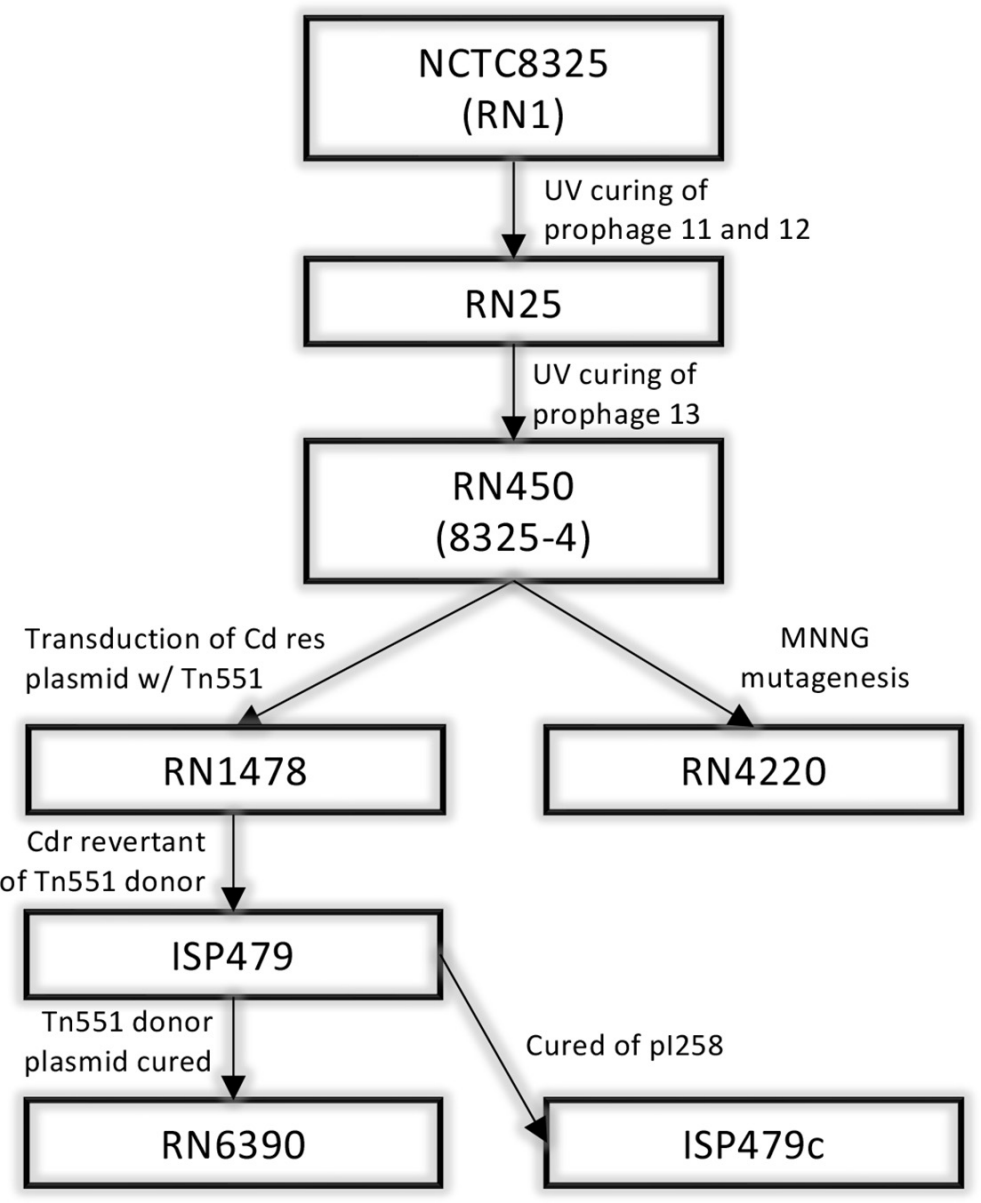
A selection of daughter strains of the NCTC8325 lineage and how they were generated. RN25 (also called 8325–3) was generated from NCTC8325 following UV curing of prophages 11 and 12 [18]. RN450 (also called 8325–4) was generated from RN25 by a second round of UV exposure, to cure prophage 13 [18]. Methylnitronitrosoguanidine (MNNG)-mediated mutagenesis of RN450 was used to generate RN4220, which is restriction deficient and capable of accepting foreign DNA [19]. RN450 was separately transduced with pRN3032, a Tn551 donor plasmid to generate RN1478 [19] ISP479 is a cadmium-resistant revertant of RN1478 [20]. RN6390 was generated from ISP479 by curing of pRN3032 [1]. The pI258 plasmid was cured from ISP479 to generate ISP479c [9]. Figure and legend adapted from [21], and removed from the subsequent publication [22].

*Staphylococcus aureus* strain RN6390 is a commonly used laboratory strain, derived from the clonal complex eight strain, NCTC8325 [1]. RN6390 has been used extensively to study the staphylococcal type VII protein secretion system (T7SS) (e.g. [2, 3]. NCTC8325 carries three prophages integrated in its genome and these were sequentially cured out of the strain using UV induction to generate the daughter strain RN450 (also known as 8325–4). RN450 has subsequently been used to generate a multitude of strains, including SH1000, RN4220 and RN6390 (Fig. 1). The genome of NCTC8325 was first sequenced by the ‘University of Oklahoma Health Sciences Center’ in 2006 and has since been used as the reference genome for many of its derivatives. Due to the extensive mutagenesis steps performed on this strain to subsequently generate RN6390, we sequenced the genome of RN6390 to assess differences in comparison to NCTC8325. We found that RN6390 carries a prophage in its genome that is unrelated to any of those in the parent NCTC8325 strain. Furthermore, the only other major differences observed between these strains were found in tandem repeating genes. A genome sequence of NCTC8325, sequenced by the Wellcome Sanger Institute on behalf of the National Collection of Type Cultures shares much greater sequence identity with RN6390, suggesting that assembly error rather than genetic changes accounts for the differences observed between RN6390 and the NCTC8325-Oklahoma sequence.

## Methods

**Fig. 2.**
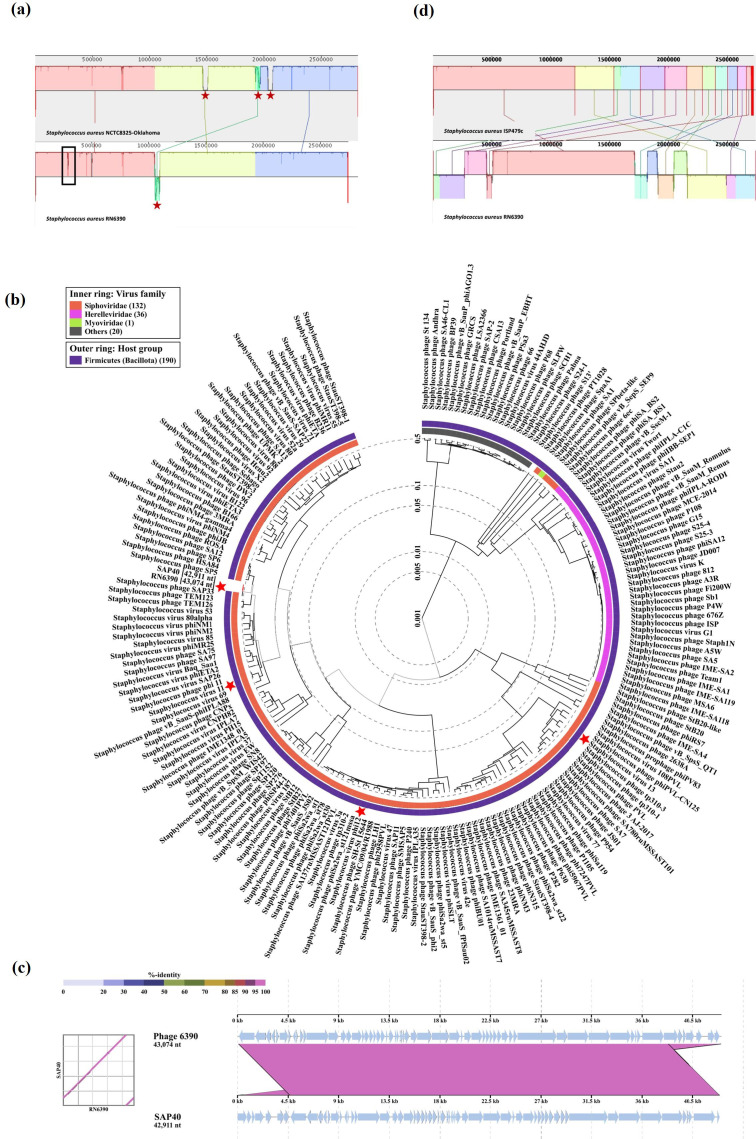
Analysis of *Staphylococcal* phage 6390. (a) Genome alignment of RN6390 (bottom) with NCTC8325-Oklahoma (top) using progressiveMauve. The black box indicates the location of the *ess/*T7 locus in RN6390. Red stars indicate the location of prophage in the genome. (b) A phylogenetic tree of Φ6390 against a database of Staphylococcal phages generated using VIPtree [6]. The positions of Φ6390 and Staphylococcal phages Φ11, Φ12 and Φ13 (excised from the NCTC8325 parent strain during construction of RN6390) on the tree are indicated with red stars. (c) Alignment of the prophage Φ6390 with SAP40 (MK801683.1) using VIPtree. (d) Genome alignment of RN6390 (bottom) with ISP479c (top) using progressiveMauve. Figure and legend adapted from [21], and removed from the subsequent publication [22].

Strain RN6390 was obtained from Professor Jan Maarten van Dijl (University of Groningen, NL). All other strains were obtained from Professor José Penadés (Imperial College, UK). All genome sequencing was carried out by MicrobesNG (Birmingham, UK) with enhanced genome sequencing (a combination of Illumina short reads with Oxford Nanopore long reads) for RN6390 and standard whole-genome service (Illumina sequencing at a minimum coverage of 30×) for RN25, RN450 and ISP479c. Genome sequences are available at NCBI under accession numbers CP090001.1, JAJSOX000000000.1, JAJSOY000000000.1 and JAMWMH000000000.1, respectively. Whole-genome alignment was executed using progressiveMauve [4]. SNP calling was carried out using Snippy v4.6.0 with default parameters [5]. To determine the taxonomy of the phage identified in RN6390 strain, its nucleotide sequence was submitted to VIPTree browser with default parameters [6]. From the resulting proteomic tree, a subset tree (Fig. 2b) was generated selecting genomes of *S. aureus* phages.

## Results

The genome of RN6390 was aligned with that of NCTC8325 sequenced by the ‘University of Oklahoma Health Sciences Center’ (hereafter called NCTC8325-Oklahoma; GenBank accession number: CP000253.1) using progressiveMauve (Fig. 2a). Three large regions are absent in RN6390 compared to NCTC8325-Oklahoma, which correspond to the three prophages, Φ11, Φ12, Φ13, that were cured out of this strain [1]. These are indicated as gaps in the coloured homology regions of the Mauve output (Fig. 2a). A large region is additionally present in RN6390 that is not found in the parent strain. This region corresponds to the prophage *Staphylococcus* phage 6390(Φ6390), integrated in the genome. Φ6390 has been previously identified in the genome of RN6390, integrated in the intergenic region between the *rpmF* and *isdB* genes [7, 8]. This is at a different locus from prophages Φ11, Φ12 and Φ13 in the NCTC8325 genome. To determine whether Φ6390 is related to any of the prophages cured from NCTC8325, the genome sequence was extracted and used to determine its taxonomy. As shown in Fig. 2b, Φ6390 is a distinct prophage to those cured from NCTC8325. Analysis of Φ6390 using blastn gave an almost identical match to *Staphylococcus* phage SAP40 (GenBank accession number: MK801683.1, 99 % coverage and 99.98 % identity) (Fig. 2c). As Φ6390 is not present in RN4220 (GenBank accession number: CP076105.1), this suggests that it was introduced during genetic manipulation steps performed following generation of RN450. To determine when Φ6390 was introduced into this lineage, we sequenced strain ISP479c, which is also derived from the RN6390 predecessor, ISP479 [9] (Fig. 1). Overall, very few differences were observed between these strains, with 13 SNPs detected and a six nucleotide deletion in a predicted transcriptional regulator gene (Fig. 2d; Table S1, available in the online version of this article). ISP479c was also found to carry Φ6390, suggesting that this phage was introduced during generation of ISP479 (Fig. 1). As transduction was used to introduce a cadmium resistance plasmid to RN450, to create RN1478, it is plausible that Φ6390 was used for the transduction of this plasmid. We were, however, unable to find a record of the transducing phage that was used for the creation of RN1478 in the literature to confirm this hypothesis.

To gain a better understanding of the differences between NCTC8325-Oklahoma and RN6390, SNP calling was performed using Snippy (Table S2). Of note, a large number of SNPs were identified in ribosomal RNA encoding genes. Upon further analysis, these SNPs appear to be due to recombination events between ribosomal RNA genes (Table S2). Similar recombination events appear to have taken place between the microbial surface components recognizing adhesive matrix molecules' (MSCRAMM) *clfB* and *sdrD* genes. Whilst this is possibly due to high homology in these genes [10, 11], it is also plausible that this phenomenon is due to sequence mis-assembly because of the highly repetitive nature of these sequences [12].

**Fig. 3.**
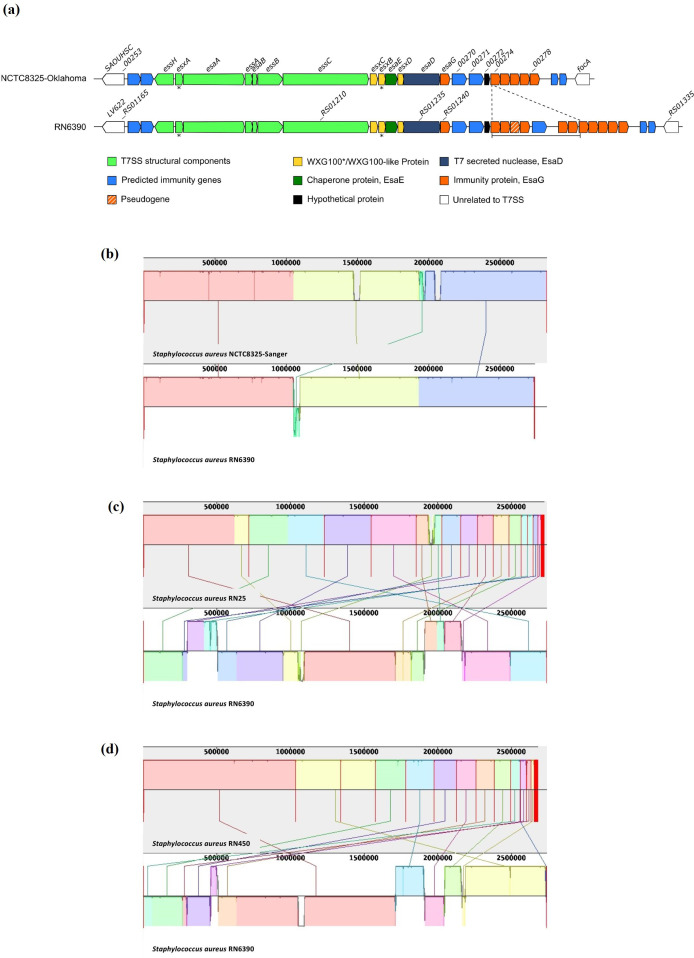
Comparison of the NCTC8325 genome to that of derived strains. (a) The genetic organization of the *ess/T7* locus in NCTC8325-Oklahoma and RN6390. The dashed lines and bar represent the region that is missing from NCTC8325-Oklahoma, as identified in this study. (b) Genome alignment of RN6390 (bottom) with NCTC8325-Sanger (top) using progressiveMauve. (c) Genome alignment of RN6390 (bottom) with RN25 (top) using progressiveMauve. (d) Genome alignment of RN6390 (bottom) with RN450 (top) using progressiveMauve. Figure and legend adapted from [21], and removed from the subsequent publication [22].

When comparing NCTC8325-Oklahoma with RN6390, a large nucleotide insertion at the *ess/T7* locus of RN6390 was identified (indicated by a black box in Fig. 2a). The T7SS in *S. aureus* secretes an antibacterial nuclease toxin, EsaD which is encoded at the T7SS locus of many *S. aureus* strains, including RN6390 [2]. To protect themselves from nuclease activity, strains also encode an immunity protein, EsaG, immediately downstream of *esaD*. However, as EsaD exists as multiple polymorphic variants across *S. aureus* strains, all strains accumulate multiple, non-identical *esaG* genes at the T7SS locus, which may protect against these toxin variants [2]. The large nucleotide insertion in RN6390 is found to increase the repertoire of *esaG* genes, from six to 12 (Fig. 3a).

A second genome sequence of NCTC8325 is also available on NCBI (hereafter called NCTC8325-Sanger; GenBank accession number: LS483365.1), which was sequenced by the Wellcome Sanger Institute, on behalf of the National Collection of Type Cultures. We aligned the genome of RN6390 to that of NCTC8325-Sanger to determine if the differences observed with NCTC8325-Oklahoma were retained. The genome of RN6390 aligned more closely to NCTC8325-Sanger than NCTC8325-Oklahoma (Fig. 3b; Fig. 2a, Table S3), and also carries the six additional *esaG* genes found in RN6390. This suggests that the genome sequence available for NCTC8325-Sanger best represents the progenitor strain of the 8325 lineage. To confirm this, we sequenced the genomes of the intermediate strains RN25 and RN450, as they are directly in the lineage of RN6390. Both of these strains share the additional copies of *esaG* and other genomic features of NCTC8325-Sanger (Fig. 3c and d), confirming that NCTC8325-Sanger more accurately represents the progenitor strain of RN6390.

## Discussion

The genome of RN6390 was sequenced and compared to the parental strain NCTC8325, for which, two genome sequences were available. RN6390 aligned more closely with NCTC8325-Sanger than NCTC8325-Oklahoma, despite NCTC8325-Oklahoma being used most commonly as a reference genome for this strain (e.g. [2, 3, 13]). NCTC8325-Sanger was constructed from long-read PacBio sequencing [14] whilst NCTC8325-Oklahoma was likely assembled from short sequence reads. Due to the repetitive nature of the ribosomal RNA genes, MSCRAMM genes and *esaG* tandem paralogues, it is possible that these differences are due to sequence assembly error from short reads, as it is difficult to assemble repetitive sequences using short reads ([12]; reviewed in [15]). However, as the reads are not available for NCTC8325-Oklahoma we cannot be certain. The genome of RN6390 in this study, was assembled using a combination of Oxford Nanopore long reads and Illumina short reads. It has been suggested that this method is the best for the sequencing of repetitive or hard to sequence genomes [16], suggesting that the sequence differences observed are likely not due to assembly errors of NCTC8325-Sanger or RN6390.

For those working on the T7SS in *S. aureus *, we would like to highlight the additional seven genes found at the *ess/T7* locus in RN6390 that were not described previously due to the use of NCTC8325-Oklahoma as a reference (e.g. [2, 3, 13, 17]). Directly downstream of *SAOUHSC_00272*, four paralogues of *esaG* are found, the third of which is annotated as a pseudogene due to the introduction of a premature stop codon in the middle of the sequence (Fig. 3a). These are followed by a DUF5079 protein-encoding gene and two further *esaG* paralogues (Fig. 3a). It should be noted that although previous work has assumed six copies of *esaG* in RN6390 [2, 3, 13], studies deleting the *esaG* cluster use *SAOUHSC_00279* to define the 3′ end of the deletion, which results in the removal of all 12 *esaG* genes and therefore does not affect the interpretation of these studies [2, 3].

## Conclusions

We have sequenced the commonly used *S. aureus* strain RN6390 to obtain a circularized genome. Comparison with the parental strain NCTC8325 confirmed that the three prophages from NCTC8325 had been cured, and that RN6390 acquired a distinct prophage as reported previously [7, 8], that has almost complete sequence identity to that of SAP40 (GenBank accession number: MK801683.1,). We have further demonstrated that the full-genome sequence of NCTC8325-Oklahoma, that is commonly used as a reference genome for NCTC8325 derivatives, has several differences within its genome, likely relating to sequence mis-assembly. These differences are predominantly in repetitive genes including ribosomal RNA genes, MSCRAMM genes such as *sdrD* and the T7SS *esaG* immunity repertoire. We recommend the use of NCTC8325-Sanger as a reference genome for NCTC8325-derivative strains.

## Supplementary Data

Supplementary material 1
